# "Official" or "Unofficial"

**Published:** 1898-02-05

**Authors:** 


### "Official" or "Unofficial."

The little squabble at the British Medical Association has blown over, and both the President of the Council and the Reference Committee have emerged triumphant. Of the two, the Reference Committee comes out by far the best—a natural consequence of the higher tone taken in answering its accusers. Dr. Saundby was apologetic, and when accused of having given official support to a particular candidate (Sir Walter Foster) for a seat on the General Medical Council, instead of maintaining his right and, in fact, his duty to act for the Association in an emergency, denied the soft impeachment, and stated that he "did not at any time entertain the thought of interfering officially in that election." There can be no doubt that this line of defence put the Council in great difficulty, for in accepting his defence they had to endorse his very wide views as to what may be legitimately regarded as "unofficial," which was rather a large order. Dr. Saundby's account of the events which took place is perfectly plain, and shows well in what a difficult position the officials of the Association found themselves placed by the perverse ingenuity of Dr. Rentoul in sending in his resignation just when he did; but it is impossible not to see that, however much Dr. Saundby may have thought he was acting unofficially, it was only by virtue of his office that he was able to act at all as he did. Under the circumstances which existed at first Dr. Saundby says that he "felt that officially there was nothing to be done but to wait." So he waited, and this action we may presume was official. Why, then, are we asked to call what he did afterwards unofficial? Briefly, the following is an account of the circumstances, and of what he did in his "unofficial" capacity: It was one o'clock on Thursday; the paper was just going to press; the nominations of candidates had to be sent in by the next Saturday at latest; it seemed out of the question to summon the Council. Dr. Saundby had just heard at the office, in answer to "our special inquiry," that Dr. Rentoul would not be allowed to withdraw his resolution resignation; so, "*thinking I was carrying out the wishes of the Association*, I asked him (Sir Walter Foster) to stand, and obtained his consent. When he, in turn, asked me to act as chairman of his election committee, *in pursuance of the same line of conduct* I agreed to do so." The President of the Council then addressed letters to the secretaries of branches, asking them to do all in their power to promote the success of Sir Walter Foster's candidature. Now in all this, according to the finding of the Council, Dr. Saundby was acting unofficially, and no doubt he was—that is to say, he had not, in the cautious words of the solicitor to the Association, "added the words, 'President of the Council' after his signature, or used paper with the Association's heading, or stated in his letter that he wrote on behalf of the Council." This, however, seems to us to be a very technical definition, and it is clear that the Council, in accepting the line of defence adopted by Dr. Saundby, has created a very serious precedent as to the liberty of official personages to perform unofficial acts pertaining to their office. The confusion that is likely to arise is almost comical to think of.

Most people will, we fancy, hold to the opinion that the acts of the President of the Council, the elect of the elected, the executive head of the Association, must be official when they have to do with Association affairs, and that minute distinctions between official and non-official action are more worthy of the Pickwick Club than of the British Medical Association.

The attack on the Reference Committee was met by Dr. Holman in a much bolder fashion. He pointed out that the Reference Committee was appointed to "guide the policy and control the contents of the journal," and that although it was quite open to the Council to criticise the policy they adopted, it was in no way open to them to complain that they had adopted a policy without reference to the Council, which was what the resolution did. No doubt both the President and the Reference Committee made a great mistake, and backed the wrong horse. Of this, however, no complaint was made. The protest was that they acted without consulting the Council, and Dr. Holman showed that this was not only absurd but *ultra vires*. Mr. Ernest Hart used to be very fond of the phrase, "grasping the nettle." Dr. Holman did as Mr. Hart would have done, he "grasped the nettle," and founded his defence on a logical position, and it would have been far better if Dr. Saundby had adopted the same course; the vote would have been the same, and he would have saved the Council from setting up a very awkward precedent.

